# Exhausted T cells and epigenetic status

**DOI:** 10.20892/j.issn.2095-3941.2020.0338

**Published:** 2020-12-15

**Authors:** Ziqing Zeng, Feng Wei, Xiubao Ren

**Affiliations:** 1 Department of Immunology, National Clinical Research Center for Cancer, Key Laboratory of Cancer Prevention and Therapy, Tianjin’s Clinical Research Center for Cancer, Key Laboratory of Cancer Immunology and Biotherapy, Tianjin 300060, China; 2 Department of Biotherapy Tianjin Medical University Cancer Institute and Hospital, National Clinical Research Center for Cancer, Key Laboratory of Cancer Prevention and Therapy, Tianjin’s Clinical Research Center for Cancer, Key Laboratory of Cancer Immunology and Biotherapy, Tianjin 300060, China

**Keywords:** T cell exhaustion, TOX, tumor immunity, epigenetic landscape, immunotherapy

## Abstract

Exhausted T cells are a group of dysfunctional T cells, which are present in chronic infections or tumors. The most significant characteristics of exhausted T cells are attenuated effector cytotoxicity, reduced cytokine production, and upregulation of multiple inhibitory molecular receptors (e.g., PD-1, TIM-3, and LAG-3). The intracellular metabolic changes, altered expression of transcription factors, and a unique epigenetic landscape constitute the exhaustion program. Recently, researchers have made progress in understanding exhausted T cells, with the definition and identification of exhausted T cells changing from phenotype-based to being classified at the transcriptional and epigenetic levels. Recent studies have revealed that exhausted T cells can be separated into two subgroups, namely TCF1^+^PD-1^+^ progenitor-like precursor exhausted cells and TCF1^-^PD-1^+^ terminally differentiated exhausted T cells. Moreover, the progenitor-like precursor cell population may be a subset of T cells that can respond to immunotherapy. Studies have also found that TOX initiates and dominates the development of exhausted T cells at the transcriptional and epigenetic levels. TOX also maintains T cell survival and may affect decisions regarding treatment strategies. In this review, we discuss the latest developments in T cell exhaustion in regards to definitions, subpopulations, development mechanisms, differences in diverse diseases, and treatment prospects for exhausted T cells. Furthermore, we hypothesize that the epigenetic state regulated by TOX might be the key point, which can determine the reversibility of exhaustion and the efficacy of immunotherapy.

## Introduction

The term “T cell exhaustion” was originally derived from a mouse chronic lymphocytic choriomeningitis virus (LCMV) infection model^1^, which is now widely used to define the dysfunctional status of T cells when stimulated by chronic infection or tumor-induced long-term high antigen burden^2^. The characteristics of T cell dysfunction include reduced cytotoxic activity, decreased ability to secrete cytokines, expression of inhibitory receptors, and reduced proliferation potential and survival rate^3^. However, some researchers question this definition and believe that the characteristics of exhausted T cells may be heterogeneous. Previous studies have shown that some exhausted T cells proliferate and have certain antitumor or antiviral effects, such as the production of cytokines or granzyme B (GZMB). Therefore, these cells can control pathogens or tumors, to some extent, and are not completely dysfunctional^2,4^. Moreover, single cell sequencing analysis has also shown that exhausted T cells express various genes related to effector functions^5–7^, confirming that exhausted T cells retain partial effector activity. In addition, other researchers have reported that some T cells present in tumors completely lose their functions, and thus, cannot prevent tumor progression. Although at the molecular level, those cells have similar characteristics to exhausted T cells during chronic infections, they should not be considered exhausted T cells, but rather dysfunctional T cells^8^. Due to these different viewpoints, we believe it is necessary to accurately summarize the latest research progress in regards to the biological characteristics of exhausted T cells.

## Definition and identification of T cell exhaustion

It is generally believed that, in the early stage of infection, naive T cells proliferate and differentiate into effector cells, which exert powerful antiviral functions. However, as the chronic infection or tumor persists, the effector T cell is stimulated by persistent antigens, and thus, gradually loses its effector functions, thereby becoming an exhausted cell^2^. This exhaustion program is useful and acts as a protective mechanism that protects the body from pathological immune damage. Notably, the functions of T cells are lost in a hierarchical manner as follows: step 1, in the early stage of exhaustion, the production of interleukin 2 (IL-2), high proliferation capacity, and *in vitro* cytotoxic function are first lost; step 2, in the intermediate stage, the ability to produce tumor necrosis factor α is lost; step 3, in the late stage of exhaustion, severe exhaustion eventually leads to the partial or complete loss of the cell’s capability to produce large amounts of interferon-gamma (IFN-γ) and GZMB; and step 4, the physical absence of T cells represents the terminal phase of T cell exhaustion. Thus, the severely exhausted T cells in the tumor microenvironment (TME) can be removed through this deletion program^2^.

### T cell exhaustion, anergy, and senescence

T cell exhaustion is significantly different from T cell anergy and senescence^9^. CD8^+^ T cell anergy indicates that the CD8^+^ T effector cells lack a second synergistic signal stimulation. Thus, although these T cells are in a survival state, they cannot proliferate or secrete IL-2. Unlike T cell exhaustion, T cell anergy may occur in naive T cells, while exhausted T cells may be produced or transformed from T cells that had been initially activated. Due to the lack of the necessary co-stimulatory signals for cell activation, anergic T cells cannot secrete cytokines, such as IL-2, and are therefore rendered ineffective much earlier. In contrast to anergy, which is rapidly developed after stimulation, exhaustion develops gradually, depending on the presence of chronic stimuli^2^. Thus, these two types of T cells occur at different stages of T cell activation. Senescent T cells are a group of terminal T cells that have lost the ability to divide and proliferate. However, they are also different from exhausted and anergic T cells. Senescent T cells have their cell cycle arrested and also present unique functions and a unique phenotype. Studies have shown that this group of cells downregulates CD27 and CD28 and upregulates CD57 and killer cell lectin-like receptor G1 (KLRG1), but does not upregulate programmed cell death 1 (PD-1) and the T-cell immunoglobulin domain and mucin domain protein 3 (TIM-3) like exhausted T cells^9^. Additionally, senescent T cells can secrete inflammatory mediators, such as IL-6 and IL-8^9^. Furthermore, exhausted T cells can reverse their exhausted state by blocking the PD-1 pathway, indicating that exhausted T cells, unlike senescent T cells, are not completely terminal cells^10^.

### Current definition of exhaustion

Currently, the identification of exhausted T cells is mainly achieved by detecting the expression of multiple inhibitory surface receptors^3,11^. Studies have suggested that T cell exhaustion severity is directly proportional to the number of inhibitory molecules co-expressed on the surface of T cells^9,12–14^. These inhibitory receptors initially included PD-1 and cytotoxic T-lymphocyte-associated protein 4 (CTLA-4). As research progressed, TIM-3, lymphocyte activation gene 3 (LAG-3), and T cell immunoreceptor with Ig and ITIM domains (TIGIT) were successively proposed as exhaustion markers^3,11,13–16^. Recent studies have indicated that there are new potential surface receptors that can be used as markers for exhausted T cells, such as CD39^17^, CD73^18^, and CD69^19^. Moreover, Chihara et al.^20^ functionally validated 2 new co-suppressor receptors, namely the protein C receptor and podoplanin. The expression of these new surface markers is often positively correlated with the expression of PD-1 and CTLA-4, and is related to a decline in effector functions, such as a decrease in the secretion of the IL-2 cytokine.

It is generally believed that the typical hallmark of T cell exhaustion is the expression of the PD-1 inhibitory receptor^12^. However, research has shown that the expression of inhibitory receptors does not accurately define the exhaustion state, as some strongly activated effector T cells may also express those receptors^16^. It was also reported that PD-1^+^ CD8 cell density is significantly positively correlated with the prognosis^21^. Moreover, even though antigen-specific T cells avoid the “exhaustion” process through transcriptional regulation and become “normal phenotype” cells, which do not express inhibitory receptors, they can still develop into functional-impaired T cells under chronic antigen stimulation. This suggests that the regulation of inhibitory receptor expression is not related to the loss of effector function^22^. Another study also reported that T cell exhaustion can occur after the genetic deletion of PD-1, as the absence of PD-1 will promote the accumulation of terminally exhausted T cell subsets^23^. There are also some T cells which express high levels of inhibitory receptors that are no longer exhausted cells, but are “used up” or dormant cells, which are entirely devoid of function. Additionally, these types of cells can also be other dysfunctional or divergent cells with an unconventional functional state, including bystander T cells and low tumor-reactive cells, which are derived from the initial phase of tumors or chronic infections^24,25^.

### A new definition of exhaustion at the transcriptional and epigenetic levels

As a unique lineage, the transcriptional definition is an important development, which refers to the method of defining cell subgroups not only by their phenotypes or functions, but also by their molecular or transcriptional characteristics. The use of single cell transcriptional and epigenetic analyses has revealed more characteristics of exhausted cells in tumors^26–28^. Although most of these characteristics are based on the identification of so-called “exhausted T cells” *via* inhibitory receptors, as most of the inhibitory receptors were found on the surface of exhausted cells, these findings may, to some extent, reflect features which belong to truly exhausted T cells. This could be of great help in revealing the possible mechanisms underlying the production and maintenance of exhausted T cells. Recent research findings have shown that, although the essential molecules characteristic of exhausted T cells have not been elucidated, exhausted T cells use completely different transcription factors and form transcription programs that differ entirely from normal memory or effector T cells^29^. The specific transcription factors related to exhaustion include NFAT, thymocyte selection-associated high-motility group (HMG) box protein (TOX), NR4A, T cell factor-1 (TCF1), Eomes, T-bet, BATF, and Blimp-1^30^, all of which play a role in promoting or sustaining the exhaustion program. These findings have also shown that exhaustion is a specific differentiation status of T cells. Thus, identifying more transcription level markers specific to exhausted T cells could help define them.

Recent epigenetic analyses have shown that the exhausted T cell subgroup differs from the effector or memory subgroups by approximately 6,000 open chromatin regions^28,31–33^, which are similar to other major hematopoietic cell lines. Those differences also suggest that exhausted T cells have a unique chromatin landscape, and are an individual cell subpopulation, rather than being transformed from memory or effector T cells. Epigenetic changes can influence PD-1 levels and help develop exhausted cells. Moreover, significant upregulations of DNA methylases were found in exhausted T cells^30,34^. Although we have yet to elucidate the epigenetic mechanism that initiates the exhaustion program, researchers have found that TOX may play an essential role in the epigenetic modeling of exhausted T cells.

There is currently no consensus on the typical hallmarks for exhausted T cells. However, rather than using the expression of inhibitory receptors or purely functional definitions, it is more accurate to define the exhausted state by using transcriptional and epigenetic features, which can be used to supplement or even replace the previous definitions^35^. Although transcriptional information is abundant, epigenetic changes are more specific and stronger^28,31,33^. Current research confirms that TOX can promote the process of exhaustion at the transcriptional and epigenetic levels. TOX may be the most critical exhaustion regulator, and its expression is an essential characteristic of exhausted T cells^22,27,36–41^. The expression level of TOX and TOX-induced molecular events or epigenetic changes may be the most accurate way to identify and quantify the T cell exhaustion status.

## The role of TOX in T cell exhaustion

TOX is a nuclear DNA binding protein. It binds to nuclear DNA in a structure-dependent manner, rather than in a sequence-dependent manner. According to previous studies, TOX plays an important role in the development of thymus CD4^+^ T cells, NK cells, and intrinsic lymphocytes^42,43^ and is critical in the differentiation of tumor-specific T cells^22^. During T cell exhaustion, TOX changes the accessibility of approximately 4,000–9,000 chromatin regions, most of which are located at the enhancer or promoter sites of critical exhaustion-related genes, thereby further influencing the expression of those genes and mediating the exhaustion program^36,44^.

In June, 2019, *Nature* and *Nature Immunology* simultaneously published 4 related articles^22,27,36,38^, describing the vital role of TOX in exhausted CD8^+^ T cell differentiation and its molecular mechanism. The 4 studies consistently found that high TOX expression levels were related to high expression of multiple inhibitory receptors and low expression of TCF1. In LCMV, hepatitis C virus, and spontaneous liver cancer models, TOX induced T cell exhaustion by promoting the expression of exhaustion-related genes and suppressing the expression of effector-related genes. Thus, it can be concluded that the transcription factor TOX is a critical regulator of exhausted T cell development in chronic infections, which influences the unique differentiation and development program of antigen-specific exhausted T cells at both the transcriptional and epigenetic levels. In acute infections, TOX is expressed only transiently and at low levels. TOX is dispensable for effector or memory T cell development, while it is essential for the formation of T cell exhaustion. Thus, exhausted T cells will not form in the absence of TOX^36^. These results further imply that the exhausted T cell is a different type of cell and not a specific activation state of the other two types of cells. TOX expression is necessary and sufficient to induce the formation of the major features of exhausted T cells. Notably, persistent high TOX expression levels are sufficient to induce all the molecular characteristics of the exhaustion program, therefore acting as a central regulator^22^. As such, TOX expression can induce the expression of inhibitory receptors and key transcription factors specific to exhaustion, decrease the secretion of functional cytokines, and initiate significant epigenetic changes in exhausted T cells^36^. The specific mechanism by which TOX promotes exhaustion will be described in detail later. Initially, TOX was induced by calcineurin and NFAT2^37^. However, after the initial expression was established, a feedforward loop was further formed. Thus, TOX expression and TOX-dependent exhausted T cell differentiation can be independent of calcineurin signals and may persist in exhausted T cells. Furthermore, TOX promotes the persistence of antigen-specific CD8^+^ T cells. The above 4 studies found that TOX expression is required for the survival of antigen-specific CD8^+^ exhausted T cells in chronic infection or cancer models. When antigens in the environment cannot be removed, TOX regulates the balance between T cell-mediated virus/tumor control and immune response, thereby preventing T cell death and immunopathology due to excessive activation. Therefore, TOX is essential for long-term CD8^+^ T cell immunity during chronic antigen stimulation. Collectively, TOX helps T cells adopt an optimal differentiation route, allowing the host to cope with persistent antigen pressure. Notably, TOX upregulation is a characteristic of both precursor exhausted cells and terminally exhausted cells (these two subtypes will be discussed in detail later), being the most representative feature of exhausted T cells throughout all stages of exhaustion. Yao et al.^27^ found a co-expressing gene module containing TOX. Compared with memory T cells, this module showed higher transcriptional activity in exhausted progenitor-like cells and was correlated with a higher abundance of active histone markers. This module was shown to be necessary for the development of progenitor-like CD8^+^ T cells. Seo et al.^37^ used a chimeric antigen receptor-engineered T cell (CAR-T) model and found that in PD-1^high^TIM-3^high^ tumor-infiltrating terminally exhausted CD8^+^ T cells, both TOX and TOX2 were significantly upregulated. Compared to wild-type and TOX or TOX2-deficient T cells, T cells with both a TOX and TOX2 deficiency were found to have stronger antitumor abilities. Similar to NR4A-deficient T cells, TOX-deficient T cells had elevated cytokine expression levels, decreased expression of inhibitory receptors, and increased access to the binding motif region associated with activation-related transcription factors. Therefore, these results indicated that TOX was essential for the T cell exhaustion-related transcriptional programs.

Immunotherapy, including PD-1/PD ligand 1 (PD-L1) blockade therapy, has been widely used and has achieved remarkable results in the treatment of various tumors^45^. However, checkpoint blockers can only partially restore T cell function. TOX and TOX-induced molecular events, such as epigenetic changes, may be responsible for the limited ability of PD-1/PD-L1 blockers to reverse T cell exhaustion. Therefore, the manipulation of TOX expression levels may be a promising treatment strategy. Studies have shown that removal of the DNA binding domain of TOX can reduce the expression of PD-1 mRNA and protein levels, increase the production of cytokines, and generate more T cells with functional phenotypes^38^. However, although TOX-deficiency in T cells initially leads to increased effector functions, it could also lead to more severe immunopathology and affect T cell survival. Eventually, the abundance of T cells will be significantly reduced, especially the subpopulation of TCF1^+^ T cells, which can self-renew^38^.

## Precursor exhausted T cells and terminally differentiated exhausted T cells

Exhausted T cells are a group of heterogeneous cells. Some studies have shown that exhausted T cells have many of the same characteristics as terminal effector T cells^10^, while other studies have shown that the effector cells, which survive the immune contraction phase, can form the exhausted cell group^46^. Recent research has suggested that exhausted T cells may include two distinct subgroups, precursor exhausted cells and terminally-differentiated exhausted cells^47^. Precursor exhausted cells have stem-like properties and can self-renew or further proliferate, as well as differentiate into terminally exhausted T cells. Thus, they can also be called exhausted progenitor cells^40,41,47^. However, terminally exhausted T cells have lost the ability to multiply and differentiate. Presently, the majority of so-called exhausted T cells refer to terminally differentiated exhausted T cells, which have limited proliferation ability, high PD-1 expression, and diminished effector functions. Moreover, their differentiation is irreversible. Recent studies have suggested that it might be useful to classify exhausted T cells into the precursor or terminal cell subgroups and distinguish them *via* the expression level of the functional marker TCF1, as the progenitor (precursor) cell population has high TCF1 expression levels^48^.

TCF1 is the downstream effector molecule of the classic Wnt signaling pathway. It plays the role of a transcription factor and histone deacetylase in humans in the development of both innate and adaptive immunities^48^. TCF1 is critical for the development of various T cell lineages. Studies have found that in the early stages of T cell differentiation, Notch signaling induces TCF1 expression^49^. TCF1 ensures the normal differentiation of T cells by upregulating genes necessary for T cell lineage development (such as GATA3, Bcl11b, and TCR components) and changing apparent characteristics^49,50^. Moreover, TCF1 is a key transcription factor for various mature T cell subpopulations, including T central memory^51^ and T follicular helper cells^52–54^. Mechanistically, TCF1 inhibits the expression of Blimp-1, TIM-3, and CISH, while promoting the expression of BCL-6, thereby increasing the amount of progenitor-like T cells^39^.

The terminally differentiated and exhausted cells are consistently derived from the TCF1^+^ exhausted precursor subpopulation^40,41,55^, which also suggests that the development trajectory of exhausted T cells may be independent of effector T cells. Precursor exhausted cells have low effector gene expression levels, but the expressions of their characteristic genes, such as *TCF1*^56^ and *BCL-6*^39^, are similar to those of the memory T cell subgroup. In addition, precursor exhausted cells express relatively fewer inhibitory receptors, and although they also express intermediate levels of PD-1, its expression in precursor exhausted cells is lower than that of terminally exhausted T cells. Moreover, they were found to have low TIM-3 expression levels and can thus be expressed as PD-1^int^TIM-3^low^TCF1^+^ cells^57^. These precursor cells exhibit stem-like and memory-like properties, and can continuously proliferate and differentiate into PD-1^hi^TIM-3^hi^TCF1^–^ terminally exhausted T cells. Details regarding the differences between these two exhausted T cell subgroups are shown in **Table 1**.

**Table 1:** Differences between precursor and terminally exhausted T cells

Features	Precursor exhausted T cells	Terminally exhausted T cells	References
Phenotype	Increased proliferation, increased longevity, polyfunctional cytokine production, can differentiate into terminally exhausted T cells	Decreased proliferation, decreased longevity, increased expression of inhibitory receptors, decreased cytokine production, comparatively high cytotoxicity	^39,58–60^
Surface markers	PD-1^int^, TIM-3^−^, CXCR5^+^, SLAMF6^+^ (Ly108^+^), CCR7^+^, LAG-3^+^, CD62L^+^, ICOS^+^	PD-1^high^, TIM-3^+^, CXCR5^−^, SLAMF6^−^, LAG-3^+^, 2B4^+^	^36,39,44,58–62^
Transcriptional profile	TCF1, BCL-6, TOX, NFAT, BATF, IRF4, EOMES, T-bet, ID3, etc.	BLIMP1, TOX, NFAT, BATF, IRF4, T-bet, ID2, RUNX, GZMB, etc.	^36,37,39,44,59–63^
Time of occurrence	Early stage of exhaustion (approximately within one week of chronic stimulation)	Late stage of exhaustion	^38,39,59,60,62^
Proliferative capacity	High	Low	^38,44,48,58–60^
Differentiation potential	High	Low	^38,44,48,58–60^
Effector function	Comparatively low (low GZMB and IFN-γ)	Comparatively high (high GZMB and IFN-γ)	^39,44,59–61,63^
Longevity	Long	Short	^39,58–60^

PD-1, programmed cell death 1; TIM-3, T-cell immunoglobulin domain and mucin domain protein 3; LAG-3, lymphocyte activation gene 3; TCF-1, T cell factor-1; TOX, thymocyte selection-associated high-motility group (HMG) box protein; GZMB, granzyme B; IFN-γ, interferon-gamma.

The subset of PD-1^+^TCF1^+^CD8^+^ progenitor-like precursor exhausted T cells in chronic infection^40,41^ or cancer^44,55^ is responsible for the persistent immune response that can be maintained during chronic antigen stimulation. Furthermore, precursor exhausted T cells are also responsible for the reactions caused by PD-1 blockers or tumor vaccines^39–41^, as they play an antiviral or antitumor role by proliferating and differentiating into terminally exhausted T cells with certain functions. Notably, the exhaustion state of terminally exhausted T cells cannot be easily reversed and they have no expansion capacity. Thus, their response to PD-1 blockers is weak and transient. As such, the antitumor immune response intensity and persistence caused by PD-1 blockers, tumor vaccines, or adoptive cell transfer^64^ may be more dependent on the number and function of TCF1^+^ precursor exhausted cells at the lesion site rather than terminally exhausted T cells. Some studies have also confirmed this hypothesis. Thus, researchers transmitted precursor exhausted cells to chronically infected or tumor-bearing mice and observed that precursor exhausted cells proliferated and differentiated into terminally exhausted T cells, as well as exerted effector functions. In contrast, with directly transmitting terminally differentiated exhausted T cells, they found that the cells hardly amplified, while also maintaining their deeply exhausted PD-1^hi^TIM-3^hi^ phenotype^39–41,55,62,65,66^. Although studies have proven that precursor exhausted T cells are critical for long-term response to checkpoint blockers in a mouse tumor model^44,55^, it is still unknown whether the PD-1^+^TCF1^+^ precursor exhausted cell population or their PD-1^+^TCF1^−^ terminally differentiated progeny or their combination is ultimately effective in controlling human tumors. It is also unclear whether their abundance or other characteristics of these two cell populations are directly related to the efficacy of immunotherapy. Although terminally differentiated exhausted T cells have a reduced effect, they may be mostly tumor-reactive. Moreover, although the poorly differentiated TCF1^+^ precursor exhausted T cells can continuously self-renew and restore vitality, most of them have a low cytotoxicity^7,42,66^. In addition, some of the precursor cells may not have tumor antigen specificity and exist only as a bystander unrelated to the tumor^7,25,41,67^.

At the epigenetic level, ATAC-seq analyses have revealed that exhausted precursor and terminally exhausted T cell subsets have partially different landscapes. The two populations of cells share some common epigenetic features, including the chromatin-accessible regions (ChARs) around the genes encoding TOX and PD-1^68^. In addition, the two populations of cells have different accessible regions from each other, which represent their unique epigenetic characteristics. Researchers found ChARs adjacent to Slamf6 specifically in precursor exhausted T cells. Moreover, in the location or enhancer/promoter regions of genes encoding cytokines, co-stimulatory cell-surface receptors, and survival/memory molecules, precursor exhausted T cells have greater chromatin accessibility. In contrast, terminally exhausted T cells have specific ChARs adjacent to Havcr2. In the regions of genes encoding co-inhibitory molecules, effector molecules and transcription factors associated with terminally exhausted T cells have greater chromatin accessibility^44^.

However, defining exhausted T cells as specific subsets may be unreasonable, as dysfunctional exhaustion is a gradual process. Perhaps progenitor-like precursor cells and terminally differentiated cells should not be considered two different states, but as a continuous developmental trajectory. Therefore, the main task is not to define markers which are characteristic for exhaustion substrates, but rather to identify the fundamental regulators of T cell exhaustion.

## When and how exhausted T cells form?

### The origin of exhausted T cells

The endogenous mechanism of T cell exhaustion has not been fully elucidated. It is still unclear from what stage of T cell development and what cell population is involved as the origin of exhausted T cells. Exhaustion may be a parallel process that can cover normal T cell differentiation, which means that T cells at any stage of differentiation could be induced into exhausted T cells and further produce descendants with exhaustion characteristics. Like many cell differentiation processes, the development of exhausted T cells may be a continuous process involving many intermediate states with distinct phenotypes and functions.

**Figure 1:**
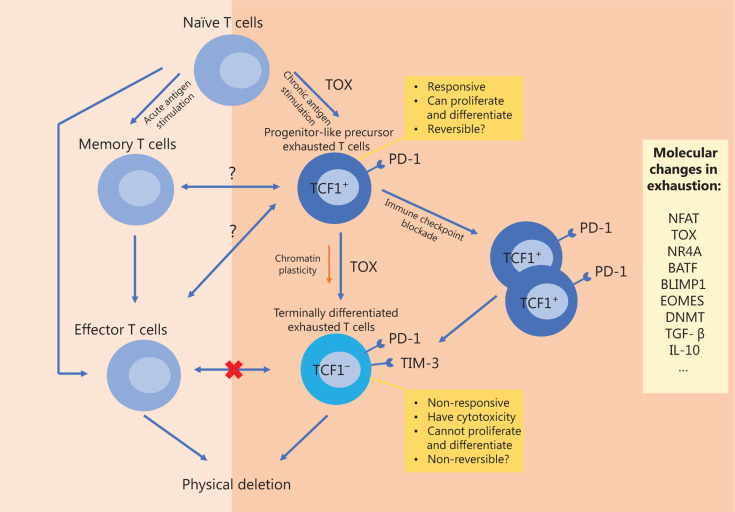
Possible developmental trajectory of exhausted T cells and the comparison and relationship with memory or effector T cells. TOX, thymocyte selection-associated high-motility group (HMG) box protein; PD-1, PD ligand 1; TCF1, T cell factor-1; TIM-3, T-cell immunoglobulin domain and mucin domain protein 3; DNMT, DNA methyltransferase; TGF-β, transforming growth factor beta; IL-10, interleukin 10.

Excessive stimulation of precursor cells may be the origin of exhausted T cells. In chronic infections or tumors, early differentiation differences emerge between memory and exhausted precursor T cells under the influence of chronic stimulation. By comparing the single cell transcriptome and epigenetic characteristics of CD8^+^ T cells that are responsive to acute or chronic viral infections, researchers have found that progenitor-like exhausted precursor CD8^+^ T cells become different from memory precursor cells prior to the response peak of T cells^27^. Among them, precursor exhausted cells further develop into terminally exhausted T cell populations^27^, a process which is similar to that by which memory precursor cells are transformed into effector T cells during acute infections^39–41,69^. In both precursor T cell populations, this expansion potential is dependent on the transcriptional regulators, TCF1, BCL-6, and FOXO1^39–41,69^. Furthermore, terminally differentiated effector T cells cannot transform into exhausted T cells^36,46^. Although the effector and exhausted cell populations are similar, both the precursor and terminally exhausted T cell populations are epigenetically or transcriptionally distinct from memory or effector T cell populations^27,28^. As previously mentioned, the TOX expression level is the most crucial differentiating characteristic of exhausted T cells, as it determines epigenetic remodeling^22,27,36–38^. The potential developmental trajectory of exhausted T cells is shown in **Figure 1**. The characteristics of exhausted T cells can be identified a few days after the appearance of a chronic infection, suggesting that exhausted cells may also occur during the early stages of infection^26^. Memory precursor T cells share many of the same characteristics as precursor exhausted T cells, suggesting the possibility of connections and transitions between these two phenotypically different populations. However, whether the exhausted (precursor) T cells arise from the transformation of normal functional T cells or differentiate *via* a completely different pathway after antigen stimulation has not yet been determined^70^. In single cell transcriptome sequencing studies of liver cancer, based on TCR analysis, researchers have found a group of T cells that appeared to be in an intermediate state between effector and exhausted T cells^71^. As such, exhausted CD8^+^ T cells are more likely to evolve from other types of CD8^+^ T cells inside the tumor. However, in a spontaneous tumor model, some researchers have proposed a new hypothesis, in which the exhausted T cell group develops from a naive T cell group, which is continuously stimulated^34^.

### Molecular biology of exhaustion programs

**Figure 2:**
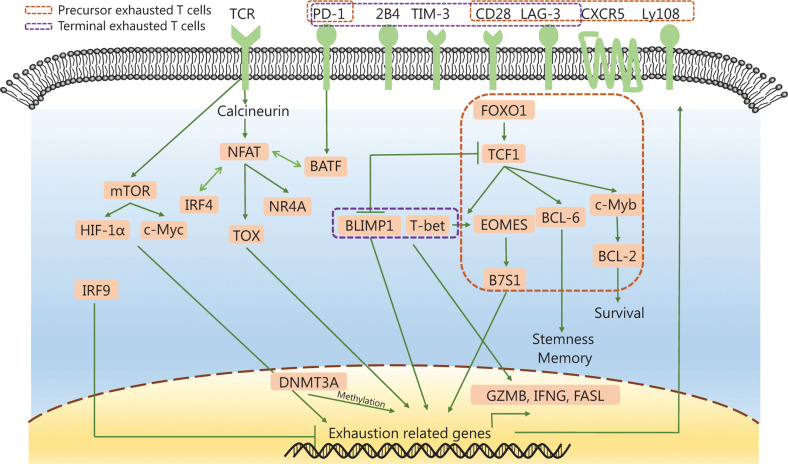
The complex intracellular regulation network of T cell exhaustion. PD-1, PD ligand 1; TIM-3, T-cell immunoglobulin domain and mucin domain protein 3; LAG-3, lymphocyte activation gene 3; TCF1, T cell factor-1; GZMB, granzyme B; TOX, thymocyte selection-associated high-motility group (HMG) box protein; DNMT3A, DNA methyltransferase 3A; IFNG, interferon gamma.

Many transcriptional, epigenetic, metabolic, and TME changes are essential mechanisms for initiating and maintaining the exhaustion program^31^. The possible regulation network of the exhaustion process is shown in **Figure 2**. Chronic TCR signaling is the core driver of exhaustion. NFAT and its downstream molecules (e.g., TOX, NR4A, and IRF4) upregulate the expression of inhibitory receptors and maintain T cell survival. In the absence of the transcription factor activator protein 1, NFAT can turn on a negative regulatory program in CD4^+^ and CD8^+^ T cells and induce low T-cell reactivity^72,73^. Moreover, the secondary transcription factors, TOX and NR4A, are both important for the CD8^+^ T cell exhaustion transcriptional programs^22,27,32,36–38,63,68,74,75^. TOX regulates the progression and maintenance of exhausted T cells^38,63^, while NR4A attenuates the antitumor effect of T cells and induces PD-1 and TIM-3 expressions^75^. Furthermore, the transcription factor, TCF1, helps in the development of stem-like properties in tumor-infiltrating lymphocytes (TILs) and supports progenitor-like exhausted T cell development. Additionally, TCF1 mediates the T-bet-to-Eomes transcription factor transition in precursor exhausted T cells by promoting Eomes expression. TCF1 can also promote the expression of c-Myb, which controls BCL-2 expression and survival^57^. Eomes overexpression promotes T cell exhaustion by B7S1 induction^76^. Notably, T-bet and Eomes oppositely influence the fate of CD8^+^ T cells^77^. B and T lymphocyte attenuator (BATF) is a downstream molecule of PD-1, which can impair T cell proliferation and cytokine secretion^78^. Blimp-1 also promotes the transformation of T cells to an exhausted phenotype during chronic infections^79^. Moreover, interferon regulatory factor 9 (IRF9) may be involved in preventing the exhaustion of T cells in tumors^80^. In terms of metabolism, mTOR upregulates the expression of inhibitory receptors in T cells by regulating the metabolic checkpoints of glycolysis *via* transcription factors, including HIF-1α and c-Myc^81^. In the TME, transforming growth factor beta (TGF-β) cytokines induce the expression of common checkpoints in TILs and inhibit the secretion of IFN-γ and GZMB^63,82^. IL-10 also inhibits IFN-γ secretion in T cells^83^.

### TOX drives multiple epigenetic enzymes to participate in exhaustion programs

The loss of function in exhausted T cells is a gradual process. At the beginning of antigen stimulation, this dysfunction is reversible. However, if antigen stimulation continues, the T cells will undergo chromatin remodeling and enter a fixed dysfunctional state, which cannot be reversed^8,31^. As previously mentioned, the transcriptional regulator, TOX, plays a key role in driving epigenetic remodeling, which occurs during exhaustion. Deletion of TOX can cause changes in chromatin accessibility in approximately 4,000 genome regions, increase chromatin accessibility at effector T cell-related gene loci, and decrease chromatin accessibility at exhausted T cell-related genes. TOX induces the epigenetic programming of exhausted T cells by recruiting and binding the histone acetyltransferase binding to ORC1 (HBO1) complexes involved in histone H4 and H3 acetylation. Studies have also reported that TOX can interact with various other chromatin remodeling proteins (Dnmt1, Leo1/Paf1, and Sap130/Sin3a)^28,31–33,36^. Other epigenetic enzymes such as Dnmt3A, Dnmt3B, and EZH2 are also involved in the exhaustion-specific programs of methylation or acetylation^34,84^. Although the dynamic cooperation of epigenetic enzymes in time and space is still unclear, by interacting with those epigenetic enzymes, TOX participates in the change of chromatin accessibility and affects the activity of various gene promoters or enhancers. In addition, TOX also changes the epigenetic landscape by regulating the transcription factor networks downstream of TCR^85^. As the opening of chromatin is closely related to the binding of specific transcription factors and the expression of corresponding genes, eventually TOX and other transcription factors form an exhaustion-specific functional status of T cells. The unique epigenetic landscape of exhausted T cells represents their completely different fate from normal effector T cells^28,31,33^.

Furthermore, TOX regulates the plasticity and reprogramming ability of T cells, which can predict the response to immunotherapy to a certain extent^31^. Once irreversible epigenetic exhaustion changes occur in T cells, adoptive transfer therapy or PD-1 blockers cannot restore T cell effector function, as they cannot produce an epigenetic reprogramming effect^28,44^. Moreover, PD-1 blockers may act by inducing stem-like precursor exhausted cells that have not undergone epigenetic changes to proliferate and differentiate into terminally exhausted T cells^28,44^. Therefore, precursor exhausted cells are the population of cells that respond after immunotherapy and are key for treatment efficacy^39–41^.

### The dynamic changes between precursor exhausted and terminally exhausted T cells

According to the enrichment of transcription initiation motifs in the ChARs, the corresponding regulatory transcription factors can be deduced. The results suggest that these two exhaustion states are maintained by the activity of different transcription factors regulated by state-specific epigenetics^44^. Based on existing results, we speculate that in precursor exhausted T cells, TOX promotes the opening of the chromatin regions that bind to transcription factors specific to precursor exhausted T cells. At the same time, FOXO1 promotes the expression of TCF1, and TCF1 promotes the expression of transcription factors such as BCL-6, EOMES, and ID3. Finally, the combination of these transcription factors with the corresponding chromatin regions promote the upregulation of CXCR5, IL-7R and other genes, making the cells show stem-like or memory-like characteristics^39,86^. With the continued existence of TCR stimulation, TOX promotes more openings in the chromatin regions that bind to transcription factors specific to terminally exhausted T cells. At the same time, IRF4 continuously activates BLIMP1 expression, and the upregulation of BLIMP1 antagonizes the effect of TCF1^79^. BLIMP1 promotes the expression of terminal-effector molecules such as GZMB, and inhibits the expression of a variety of memory-like cell-related genes^79^. Meanwhile, the differentiation regulators of effector T cells (such as T-bet, ID2) gradually replace EOMES and ID3 and gain a dominant position^47,87^. Finally, the above changes promote the transition of exhausted T cells to a terminally differentiated state.

## Differences in exhaustion between chronic infections and tumors

In different diseases or species, the core molecular processes of exhausted cells are shared. However, exhaustion also has specific characteristics in different situations^22^.

The origin of T cell exhaustion in both chronic infections and tumors is still controversial. However, the two may differ mainly due to differences in auxiliary signals, and these differences may exist even between different TMEs^34,44^. Chronic infections are an environment inundated with inflammation, in which naive T cells can undergo antigen-mediated initial activation and develop into effector cytotoxic T lymphocytes (CTLs). Subsequently, due to the failure to remove antigens, effective CTLs are exhausted, but retain some effector functions. Studies have revealed that eliminating exhausted T cells in chronic infections causes rapid progression of the infection^26^. However, unlike in chronic infections, T cells are fully activated and subsequently converted to exhausted T cells that retain some functions. In the process of tumor progression, the first carcinogenic incident is followed by a more extended incubation stage. The low expression and low immunogenicity of tumor antigens lead to an insufficient presentation of tumor antigens to T cells. Therefore, some researchers believe that the exhausted T cells in tumors develop directly from naive T cells and that there is no stable exhausted progenitor cell population like that found in chronic infections. Moreover, unlike the chronic infection environment, the TME after tumor formation is a more complex immunosuppressive environment. Compared with exhausted CTLs in chronic infections, T cells infiltrating in the TME will encounter a more complex regulatory network with various components, including cancer cells, inflammatory cells, and stromal cells, as well as various cytokines. Altogether, these factors strongly promote the conversion of T cells to an exhausted phenotype^44^.

## How to regulate the exhaustion process and the clinical application prospects for exhausted T cells

T cell exhaustion is a phenomenon that is widely observed in humans. Similar to negative immune regulators, T cell exhaustion helps the body establish immune balance under the stimulation of chronic inflammation and limits T cell-mediated immunopathology. This adaptation mechanism can prevent damage to the human body caused by excessive immune activation^38,88,89^. Moreover, even if T cells have a reduced function in their exhausted state, they still retain a certain effector capacity, ensuring a certain degree of virus or tumor control.

However, the exhaustion program of T cells is also utilized by tumors. By inducing exhaustion, the antitumor effect of CTLs is greatly restricted, resulting in uncontrolled tumor growth and metastasis. As immune cells are a vital antitumor force in the human body, immunotherapy technologies have been developed and have revolutionized tumor treatment strategies. These immunotherapies include immune checkpoint blockers, adoptive transfer of TILs, TCR-engineered T cells (TCR-T), and CAR-T. However, these immunotherapies are hampered by the presence of T cell exhaustion. For example, TILs or CAR-Ts express high levels of PD-1, TIM-3, or CTLA-4 after infiltrating into the tumor, indicating that they have already become exhausted T cells^90^. In addition, the role of immune checkpoint inhibitors is very dependent on endogenous T cell functions. However, they cannot reverse T cell exhaustion in cells that have undergone epigenetic changes. As such, this limits the long-term efficacy and widespread application of cancer immunotherapies. At present, the FDA-approved treatments targeting exhaustion include six PD-1/PD-L1 inhibitors and one CTLA-4 inhibitor. However, only ˜20% patients respond to these treatments^91,92^. Universal biomarkers have not been found to accurately predict the reactivity of checkpoint inhibitors; therefore, we cannot accurately screen target patients who may benefit. Other agents that target T cell exhaustion are still under laboratory research or clinical trials, so their efficacy is not clear. The development of agents targeting epigenetic changes has not yet been widely conducted. Interestingly, in a study using a mouse model of ovarian cancer, the inhibitors of DNA methyltransferase and histone deacetylase reduced the immunosuppression of the tumor microenvironment and improved the response to anti-PD-1 therapy^93^.

With the help of single cell transcriptome sequencing and epigenetic analysis, researchers have discovered many signaling pathways and controllable checkpoints that produce and maintain T cell exhaustion. By targeting these regulatory points, we may be able to preemptively influence the formation and development of exhausted cells^70^. However, we do not know how and if to regulate the process of T cell exhaustion to enable the efficient use of T cell’s antitumor effects. Moreover, is it feasible to perform a reversal of the exhausted state to an effector state to a certain degree, and will there be adverse side effects? Would it be better if we focus on increasing the abundance of stem-like precursor exhausted cells? Studies have shown that checkpoint blockers can, at least temporarily, restore the effector function of exhausted T cells in tumors^67^. However, the long-lasting therapeutic effects depend on the presence of stem-like precursor exhausted cells or early stage exhausted T cells^7,44^, whose presence and abundance may also be predictors of the patient’s prognosis. Adoptive transfer therapy cells (such as CAR-T) can be designed to be exhaustion resistant^94^ or to maintain their stem-like characteristics^95^ through gene-editing technology^33^ before infusion into the human body.

The biggest challenge of tumor immunotherapy may be the irreversible epigenetic changes present in exhausted T cells^35^. Different epigenetic states determine the remodeling ability of T cells. A more plastic chromatin state in the early stages means a greater ability to respond to the reprogramming induced by immunotherapy, while a more solid epigenetic state is resistant to reprogramming^31^. Although PD-1 blockers bring temporary changes in transcription levels and functions, once epigenetic fixation of advanced exhausted T cells forms, these reactivated T cells remain exhausted and their response to treatment is temporary and cannot be maintained^28^. As such, probably due to the fact that precursor exhausted cells are in the early stage of epigenetic changes, they are more responsive to PD-1 blockers. Preventing epigenetic fixation may be a method to reverse T cell exhaustion, but this may damage their survival or other T cell functions^35^. Moreover, there is no mature treatment technology for cell dedifferentiation. Recent progress in regards to the crucial role of TOX in exhaustion suggests that TOX may be the key “set-point” that can reverse the epigenetic and functional changes in exhausted T cells. However, the TOX gene plays a dual role. On the one hand, TOX promotes immune exhaustion, and on the other hand, TOX can be a critical factor for the long-term survival and maintenance of T cells^38^. The exhaustion-driven suppression program itself is used to prevent terminal differentiation and apoptosis of T cells caused by excessive activation. Therefore, the absence of genes related to exhausted cells might eventually promote the apoptosis of T cells^22,36,38^. Studies have shown that deletion of genes encoding PD-1, TOX, or IRF4 can reduce the survival rate of exhausted T cells and effector T cells, and ultimately reduce the number of cells in the antigen-specific T cell pool^22,23,36,38,96^. As a result, tumor progression is no longer controlled^22^. Moreover, the immunopathology caused by excessive stimulation resulting from the removal of TOX is also worthy of caution^38^. Therefore, the regulation of exhaustion factors such as TOX should be done cautiously and in a gradual manner. Furthermore, when the majority of CTLs within the tumor are epigenetically fixed and non-plastic, the role of checkpoint blockers may not be to reverse the function of existing exhausted CTLs, but rather to recruit new functional or reversible T cells to the tumor site directly from peripheral blood and prevent them from developing an exhausted phenotype.

In summary, considering the important role of epigenetic changes in the development of exhaustion, we can try to combine inhibitors of exhaustion-related epigenetic enzymes with checkpoint inhibitors in tumor therapy. Hopefully, we can partially reverse the epigenetic program of exhaustion. In the future, we need to better understand the process and critical regulatory factors involved in T cell exhaustion in order to inhibit or reverse exhaustion without damaging the positive effects of T cells. As such, stem-like TCF1^+^ precursor exhausted cells that can proliferate and self-renew may provide a breakthrough in cancer therapy^44,55^.

## Conclusions

In conclusion, exhausted T cells are a group of dysfunctional T cells present in chronic infections or tumors, with characteristics such as reduced effector cytotoxicity, reduced cytokine production, and upregulation of inhibitory receptors. Multiple metabolic, transcriptional, and epigenetic changes are involved in the exhaustion program, and the definition and identification of exhausted T cells are no longer done by the phenotype, but at the transcriptional and epigenetic levels. Recent studies have revealed the existence of two subgroups of exhausted cells: TCF1^+^PD-1^+^ progenitor-like precursor exhausted T cells and TCF1^-^PD-1^+^ terminally differentiated exhausted T cells. Studies have also found that TOX initiates and dominates the development of exhausted T cells at the transcriptional and epigenetic levels. TOX also maintains T cell survival and may affect decisions regarding treatment strategies. Although it is still unclear how we can reverse T cell exhaustion, it is believed that at least a part of exhausted T cells could be reversed to respond to checkpoint blockers. Finally, we hypothesize that the epigenetic state regulated by TOX might be the essential point that determines the reversibility of exhaustion and the efficacy of immunotherapy.
